# Sepsis with liver dysfunction and coagulopathy predicts an inflammatory pattern of macrophage activation

**DOI:** 10.1186/s40635-022-00433-y

**Published:** 2022-02-21

**Authors:** Renee R. Anderko, Hernando Gómez, Scott W. Canna, Bita Shakoory, Derek C. Angus, Donald M. Yealy, David T. Huang, John A. Kellum, Joseph A. Carcillo, For the ProCESS Investigators, Derek C. Angus, Amber E. Barnato, Tammy L. Eaton, Elizabeth Gimbel, David T. Huang, Christopher Keener, John A. Kellum, Kyle Landis, Francis Pike, Diana K. Stapleton, Lisa A. Weissfeld, Michael Willochell, Kourtney A. Wofford, Donald M. Yealy, Erik Kulstad, Hannah Watts, Arvind Venkat, Peter C. Hou, Anthony Massaro, Siddharth Parmar, Alexander T. Limkakeng, Kori Brewer, Theodore R. Delbridge, Allison Mainhart, Lakhmir S. Chawla, James R. Miner, Todd L. Allen, Colin K. Grissom, Stuart Swadron, Steven A. Conrad, Richard Carlson, Frank LoVecchio, Ednan K. Bajwa, Michael R. Filbin, Blair A. Parry, Timothy J. Ellender, Andrew E. Sama, Jonathan Fine, Soheil Nafeei, Thomas Terndrup, Margaret Wojnar, Ronald G. Pearl, Scott T. Wilber, Richard Sinert, David J. Orban, Jason W. Wilson, Jacob W. Ufberg, Timothy Albertson, Edward A. Panacek, Sohan Parekh, Scott R. Gunn, Jon S. Rittenberger, Richard J. Wadas, Andrew R. yEdwards, Matthew Kelly, Henry E. Wang, Talmage M. Holmes, Michael T. McCurdy, Craig Weinert, Estelle S. Harris, Wesley H. Self, Carolyn A. Phillips, Ronald M. Migues

**Affiliations:** 1 Department of Critical Care Medicine, University of Pittsburgh, Pittsburgh, PA USA; 2 Center for Critical Care Nephrology, Department of Critical Care Medicine, University of Pittsburgh, School of Medicine, 3550 Terrace Street, 6th floor Scaife Hall, Pittsburgh, PA 15261 USA; 3 RK Mellon Institute and Pediatric Rheumatology, Children’s Hospital of Pittsburgh of University of Pittsburgh Medical Center, Pittsburgh, PA USA; 4 Present Address: Division of Rheumatology, The Children’s Hospital of Philadelphia, Philadelphia, PA USA; 5 Translational Autoinflammatory Disease Studies Unit, Laboratory of Clinical Immunology and Microbiology, National Institute of Allergy and Infectious Diseases, Bethesda, MD USA; 6 Department of Emergency Medicine, University of Pittsburgh, Pittsburgh, PA USA

**Keywords:** Sepsis, Ferritin, IL-18, Phenotype, Organ dysfunction

## Abstract

**Background:**

Interleukin-1 receptor antagonists can reduce mortality in septic shock patients with hepatobiliary dysfunction and disseminated intravascular coagulation (HBD + DIC), an organ failure pattern with inflammatory features consistent with macrophage activation. Identification of clinical phenotypes in sepsis may allow for improved care. We aim to describe the occurrence of HBD + DIC in a contemporary cohort of patients with sepsis and determine the association of this phenotype with known macrophage activation syndrome (MAS) biomarkers and mortality. We performed a retrospective nested case–control study in adult septic shock patients with concurrent HBD + DIC and an equal number of age-matched controls, with comparative analyses of all-cause mortality and circulating biomarkers between the groups. Multiple logistic regression explored the effect of HBD + DIC on mortality and the discriminatory power of the measured biomarkers for HBD + DIC and mortality.

**Results:**

Six percent of septic shock patients (*n* = 82/1341) had HBD + DIC, which was an independent risk factor for 90-day mortality (OR = 3.1, 95% CI 1.4–7.5, *p* = 0.008). Relative to sepsis controls, the HBD + DIC cohort had increased levels of 21 of the 26 biomarkers related to macrophage activation (*p* < 0.05). This panel was predictive of both HBD + DIC (sensitivity = 82%, specificity = 84%) and mortality (sensitivity = 92%, specificity = 90%).

**Conclusion:**

The HBD + DIC phenotype identified patients with high mortality and a molecular signature resembling that of MAS. These observations suggest trials of MAS-directed therapies are warranted.

**Supplementary Information:**

The online version contains supplementary material available at 10.1186/s40635-022-00433-y.

## Background

Driven by excessive cellular activation and cytokine overproduction, macrophage activation syndrome (MAS) is an acute hyperinflammatory state characterized by hyperferritinemia, coagulopathy, and hepatobiliary dysfunction [1–4]. The associated fulminant cytokine storm results in rapidly progressing organ dysfunctions and early death without appropriate therapies. MAS is a form of secondary hemophagocytic lymphohistiocytosis (sHLH) often observed in patients with underlying rheumatic diseases [5], but this syndrome can also arise in a subset of patients with sepsis [6].

Although earlier recognition and increasing compliance to best practices have reduced in-hospital mortality from sepsis over the past decade, high profile clinical trials targeting specific inflammatory pathways have yet to improve survival, as most trial designs work on a presumption that sepsis behaves homogeneously despite presenting as a heterogenous syndrome [7–11]. Consequently, the identification of subgroups of patients who may benefit from specific biological response modifiers represents a strategic opportunity to overcome current limitations and failures [12].

Illustrating this point, in a post-hoc analysis of a randomized clinical trial investigating the effect of anakinra (a recombinant IL-1 receptor antagonist) on mortality in sepsis [9], Shakoory et al. demonstrated that anakinra, compared with placebo, resulted in a 50% relative risk reduction in mortality only in the subset of sepsis patients also presenting with features of MAS [13].

The need for prompt, specific treatment with anakinra requires early recognition, but guidelines developed to facilitate the diagnosis of MAS are impractical, because sepsis patients often present without the classic MAS features. Therefore, the clinical phenotype of concomitant hepatobiliary dysfunction and disseminated intravascular coagulation (HBD + DIC) proposed by Shakoory et al. has been used as a more practical strategy for identifying sepsis patients who may respond to anakinra [13–16]. Despite its simplicity, it remains unclear whether patients with HBD + DIC truly represent a subgroup of patients with an inflammatory pathophysiology similar to that of MAS.

We sought to describe the frequency of HBD + DIC in a contemporary cohort of patients with septic shock. We also hypothesized that HBD + DIC would be (i) an independent risk factor for mortality and (ii) distinguished by a biomarker signature associated with macrophage activation.

## Methods

### Study design, population, and setting

We conducted a nested case–control study using de-identified plasma samples and data sets from patients enrolled in the Protocol-Based Care for Early Septic Shock (ProCESS) cohort, approved by the University of Pittsburgh’s Institutional Review Board (PRO16070600). ProCESS enrolled 1341 adult patients from 31 hospitals in the United States between 2008 and 2013. Patients in the emergency department with suspected septic shock were deemed eligible if they met at least two systemic inflammatory response syndrome criteria and had either refractory hypotension or evidence of poor perfusion. Patients randomly received one of three resuscitation strategies: (i) protocol-based early goal directed therapy, (ii) protocol-based standard therapy that did not require placement of a central venous catheter, or (iii) usual care [17].

### Definitions, study cohort, and data collection

We assessed the presence of HBD + DIC based on criteria used in prior studies [13–16]. HBD was defined as a liver Sequential Organ Failure Assessment (SOFA) score ≥ 1 (i.e., total bilirubin ≥ 1.2 mg/dL), and DIC as a platelet count ≤ 100 × 10^9^/L and an international normalized ratio (INR) ≥ 1.5 IU. Cases and controls were both drawn from the ProCESS cohort, allowing for the matching of all patients based on the inclusion and exclusion criteria of the clinical trial, with an equal number of controls selected at random using age as the sole matching criterion. This was due to age being the primary confounder for survival analysis.

### Sample collection and human plasma biomarker assays

The ProCESS trial team collected whole blood using EDTA or lithium heparin as an anticoagulant within 30 min of randomization (0 h) [17]. Stored plasma samples were used to quantify the 26 biomarkers investigated in this study, selected based on their role in macrophage activation and/or their association with MAS subsets [1–4, 13, 18–36] (Additional file 1: Table S1).

Personnel performing the biomarker measurements were blinded to patient cohort and outcome. EDTA plasma samples were diluted 25-fold for the measurement of IL-18, IL-18BP, and CXCL9 as described previously [34]. The remaining analytes, with the exception of ferritin, were quantified from EDTA plasma samples diluted fourfold as part of the Human Bio-Plex Pro™ Human Inflammation Panel 1 or Cytokine Group I/II assays (Bio-Rad; Hercules, CA) per the manufacturer’s instructions. Concentrations obtained using the Bio-Plex® MAGPIX™ Multiplex Reader were normalized to the same standards, and an offset (equal to the average difference in controls between plates) was added to each value to minimize batch effects and values close to the lower limit of detection. Human ferritin levels were analyzed from heparinized plasma collected at 0 h using the Beckman Coulter UniCel DxI 800. The tests were performed by the University of Pittsburgh Medical Center (UPMC) Presbyterian Automated Testing Laboratory (Pittsburgh, PA).

### Outcomes

The primary outcome was 90-day all-cause mortality. Secondary outcomes included in-hospital mortality, individual differences in the selected biomarkers between cases and controls, and the ability of the biomarker panel to identify HBD + DIC in sepsis patients and to predict 90-day mortality in those with the HBD + DIC phenotype.

### Statistical analysis

Data were analyzed using Prism version 9.1.2 (GraphPad; San Diego, CA). Statistical significance was determined for categorical measures by Fisher’s Exact test, with the Freeman–Halton extension where applicable, and for continuous, non-normally distributed measures by the Mann–Whitney *U* test, with correction for multiple comparisons using the Holm–Šídák method. Linear relationships were determined by Spearman correlation analysis. Receiver operating characteristic (ROC) curves created for each analyte were evaluated for their individual ability to predict HBD + DIC. Multiple logistic regression analysis assessed the discriminatory power of the measured biomarkers for HBD + DIC and 90-day mortality, and we used the log-rank (Mantel–Cox) test for survival comparisons. To generate the heatmap (Partek® Genomics Suite® 6.6), we standardized values at time 0 h for each of the analytes by shifting to a median of zero and scaling to a standard deviation of one. Analyte hierarchical clusters (rows) used Euclidean distance as the measure of dissimilarity and the columns were subjected to forced clustering by group (i.e., sepsis controls and HBD + DIC). In the case of missing data, individuals were excluded from analysis for that particular variable. Missing data are enumerated in their respective tables and figures, as well as in Additional file 1: Table S2.

### Sensitivity analysis

A potential limitation of using HBD as a criterion for the identification of cases is that liver SOFA scores conflate chronic and acute liver disease. Therefore, we performed a sensitivity analysis to determine whether (i) the biomarker signature, (ii) 90-day mortality, and (iii) the risk of death at 90 days were different between patients with and without chronic liver disease in the HBD + DIC subset. In addition, as the matching of patients with HBD + DIC to controls occurred only based on age and septic shock, we performed multiple logistic regression analysis to determine whether HBD + DIC was an independent factor associated with mortality in the presence of other potentially confounding variables, which included age, race, gender, ProCESS resuscitation strategy, Charlson Comorbidity Index, Acute Physiology and Chronic Health Evaluation (APACHE) III score, creatinine level, and white blood cell (WBC) count. These confounders were specifically chosen for their inherent association with disease severity and/or mortality in sepsis [14, 37–43].

## Results

### *Patient characteristics and distribution of the HBD* + *DIC phenotype in a contemporary septic shock cohort*

Of the 1341 individuals enrolled in the ProCESS trial [17], 35% (*n* = 465) had HBD and 16% (*n* = 211) had DIC. Only 6.1% (*n* = 82) of septic shock patients presented with concomitant HBD + DIC, thereby constituting our cases. Notably, the prevalence of HBD + DIC was similar in each of the treatment arms of the ProCESS trial, with cases distributed comparably across the three resuscitation strategies (Additional file 1: Fig. S1A). In addition to having lower total bilirubin levels, higher platelet counts, and lower liver and coagulation SOFA scores, controls differed from the remaining ProCESS patients without HBD + DIC (*n* = 1177) in gender and in the proportion self-identifying as ‘other race’. However, in-hospital and 90-day mortality were similar (Table 1). Compared with sepsis controls, HBD + DIC patients had lower WBC counts but higher creatinine levels and APACHE III scores. The HBD + DIC cohort was also characterized by a higher Charlson Comorbidity Index, with an increased prevalence of chronic liver disease, renal impairment, and cancer (Table 1).

**Table 1:** Clinical characteristics of septic shock patients from the ProCESS cohort

	ProCESS^a^ *n* = 1177	Sepsis controls *n* = 82	HBD + DIC *n* = 82	*p* value all vs ctrl	*p* value ctrl vs HBD + DIC
Demographics					
Age	62, 51–74	59, 47–73	61, 48–70	0.51	0.53
Gender, Male, *n* (%)	641 (54)	55 (67)	52 (63)	0.029	0.74
Race, White, *n* (%)	807 (69)	52 (63)	52 (63)	0.33	> 0.99
Race, Black/African American, *n* (%)	297 (25)	20 (24)	18 (22)	> 0.99	0.85
Race, Asian, *n* (%)	21 (2)	2 (2)	3 (4)	0.66	> 0.99
Race, American Indian/Native Alaskan, *n* (%)	3 (0.3)	1 (1)	1 (1)	0.24	> 0.99
Race, Native Hawaiian/Pacific Islander, *n* (%)	5 (0.4)	0 (0)	1 (1)	> 0.99	> 0.99
Race, Unknown, *n* (%)	11 (0.9)	1 (1)	1 (1)	0.56	> 0.99
Race, Other, *n* (%)	33 (3)	6 (7)	6 (7)	0.037	> 0.99
Ethnicity, Hispanic, *n* (%)	121 (10)	9 (11)	13 (16)	0.85	0.49
Laboratory values (0 h)					
WBC (10^9^/L) (*n*)	14.2, 8.4–20.4 (*1172*)	15.0, 10.9–22.0 (*82*)	9.7, 4.7–18.9 (*82*)	0.101	0.001
Bilirubin (mg/dL) (*n*)	0.9, 0.6–1.5 (*1092*)	0.6, 0.4–0.9 (*75*)	3.1, 1.8–5.4 (*82*)	< 0.001	< 0.001
Platelets (10^9^/L) (*n*)	209, 146–288 (*1148*)	251, 188–327 (*82*)	49, 32–77 (*82*)	< 0.001	< 0.001
Creatinine (mg/dL) (*n*)	1.6, 1.1–2.6 (*1157*)	1.6, 1.0–3.1 (*81*)	2.2, 1.4–3.3 (*82*)	0.83	0.024
INR (IU) (*n*)	1.3, 1.2–1.6 (*959*)	1.2, 1.1–1.5 (*67*)	1.7, 1.5–2.2 (*82*)	0.026	< 0.001
Clinical Scores and Interventions (0 h)					
Hemodynamic SOFA	1, 1–4	1, 1–4	3.5, 1–4	0.66	0.059
Respiratory SOFA	1, 1–3	2, 1–3	1, 1–3	0.184	0.59
Central Nervous System SOFA	0, 0–1	0, 0–1	0, 0–1	0.38	0.032
Coagulation SOFA	0, 0–1	0, 0–0	3, 2–3	0.001	< 0.001
Liver SOFA	0, 0–1	0, 0–0	2, 1–2	< 0.001	< 0.001
Renal SOFA	1, 0–2	1, 0–2	2, 1–3	0.66	0.016
APACHE III Score^b^	57, 42–72	52, 41–65	69, 51–85	0.143	< 0.001
Mechanical Ventilation, *n* (%)	207 (18)	18 (22)	10 (12)	0.30	0.145
Comorbidities					
Charlson Comorbidity Index^c^	2, 1–4	2, 1–3	4, 2–7	0.98	0.001
Hypertension, *n* (%)	697 (59)	51 (62)	41 (50)	0.64	0.157
Myocardial Infarction, *n* (%)	127 (11)	4 (5)	12 (15)	0.094	0.063
Comorbidities (continued)					
Congestive Heart Failure, *n* (%)	144 (12)	7 (9)	10 (12)	0.38	0.61
Chronic Respiratory Disease, *n* (%)	262 (22)	21 (26)	15 (18)	0.49	0.35
Cerebral Vascular Disease, *n* (%)	107 (9)	16 (20)	3 (4)	0.006	0.003
Peripheral Vascular Disease, *n* (%)	97 (8)	6 (7)	7 (9)	> 0.99	> 0.99
Diabetes Mellitus, *n* (%)	399 (34)	34 (41)	25 (30)	0.186	0.193
Chronic Liver Disease, *n* (%)	76 (6)	4 (5)	31 (38)	0.81	< 0.001
Renal Impairment^d^, *n* (%)	172 (15)	14 (17)	27 (33)	0.52	0.029
AIDS, *n* (%)	33 (3)	1 (1)	4 (5)	0.72	0.37
Cancer^e^, *n* (%)	194 (16)	10 (12)	30 (37)	0.36	0.001
Mortality					
In-hospital, *n* (%)	215 (18)	8 (10)	35 (43)	0.052	< 0.001
90 days, *n* (%)	332 (28)	19 (23)	46 (56)	0.37	< 0.001

Data expressed as median with interquartile range unless otherwise noted

Complete data sets were available for each variable with the exception of those listed under *Laboratory Values*. The number of data points analyzed for each variable are noted for each group

APACHE, acute physiology and chronic health evaluation; ctrl, sepsis controls; HBD + DIC, sepsis with hepatobiliary dysfunction and disseminated intravascular coagulation; INR, international normalized ratio; IU, international units; SOFA, sequential organ failure assessment; WBC, white blood cells

^a^Represents the entire ProCESS cohort (*n* = 1341) minus the cases and controls included in the present study

^b^Scores on the APACHE III range from 0 to 299, with higher scores indicating greater severity of illness

^c^The Charlson Comorbidity Index measures the effect of coexisting conditions on mortality, with scores ranging from 0 to 33 and higher scores indicating a greater burden of illness

^d^Defined as history of chronic renal disease or Blood Urea Nitrogen (BUN) greater than 40 mg/dL and creatinine greater than 2 mg/dL

^e^Active at the time of enrollment or diagnosed within 1 year prior to enrollment

### *HBD* + *DIC is an independent risk factor for death during sepsis*

**Fig. 1:**
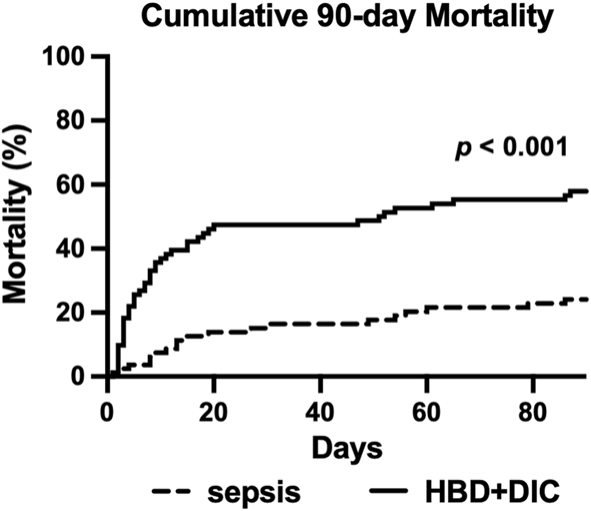
HBD + DIC phenotype in sepsis is marked by higher mortality. Unadjusted Kaplan–Meier curve comparing cumulative 90-day mortality in sepsis controls vs sepsis with HBD + DIC

In comparison with controls, HBD + DIC patients experienced higher in-hospital (43% vs 10%, *p* < 0.001) and 90-day mortality (56% vs 23%, *p* < 0.001), as would be expected given their higher APACHE III score and Charlson Comorbidity Index (Table 1; Fig. 1; Additional file 1: Fig. S1B). However, HBD + DIC was an independent risk factor for 90-day mortality in the nested case–control cohort after adjusting for these and other clinically relevant confounding variables related to disease severity and mortality in sepsis (*n* = 163) (OR = 3.1, 95% CI 1.4–7.5, *p* = 0.008). We further confirmed HBD + DIC as an independent risk factor for 90-day mortality in the entire ProCESS cohort (*n* = 1315) (OR = 2.5, 95% CI 1.5–4.3, *p* = 0.001). In the ProCESS trial, no significant differences in primary or secondary outcomes were reported between resuscitation strategy groups [17]; likewise, 90-day mortality was not affected by treatment protocol within our cohort of matched cases and controls (Additional file 1: Fig. S1C).

### *The HBD* + *DIC phenotype has a biomarker profile resembling that of MAS*

**Fig. 2:**
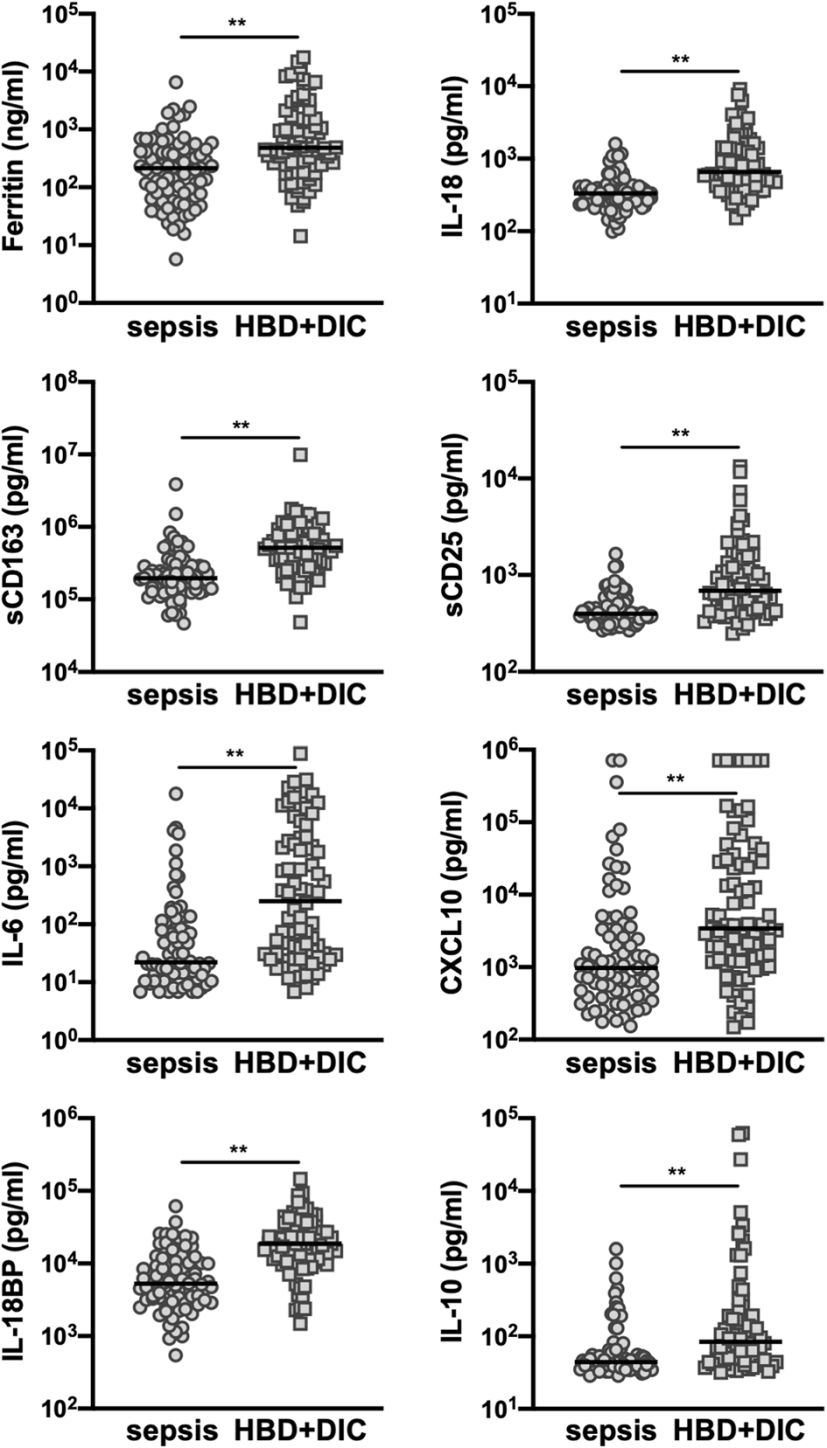
Head-to-head comparison of 8 of the 26 biomarkers between sepsis controls and sepsis with HBD + DIC. The comparison was achieved using the Mann–Whitney *U* test. **p < 0.01 after correction for multiple comparisons using the Holm-Šídák method

**Fig. 3:**
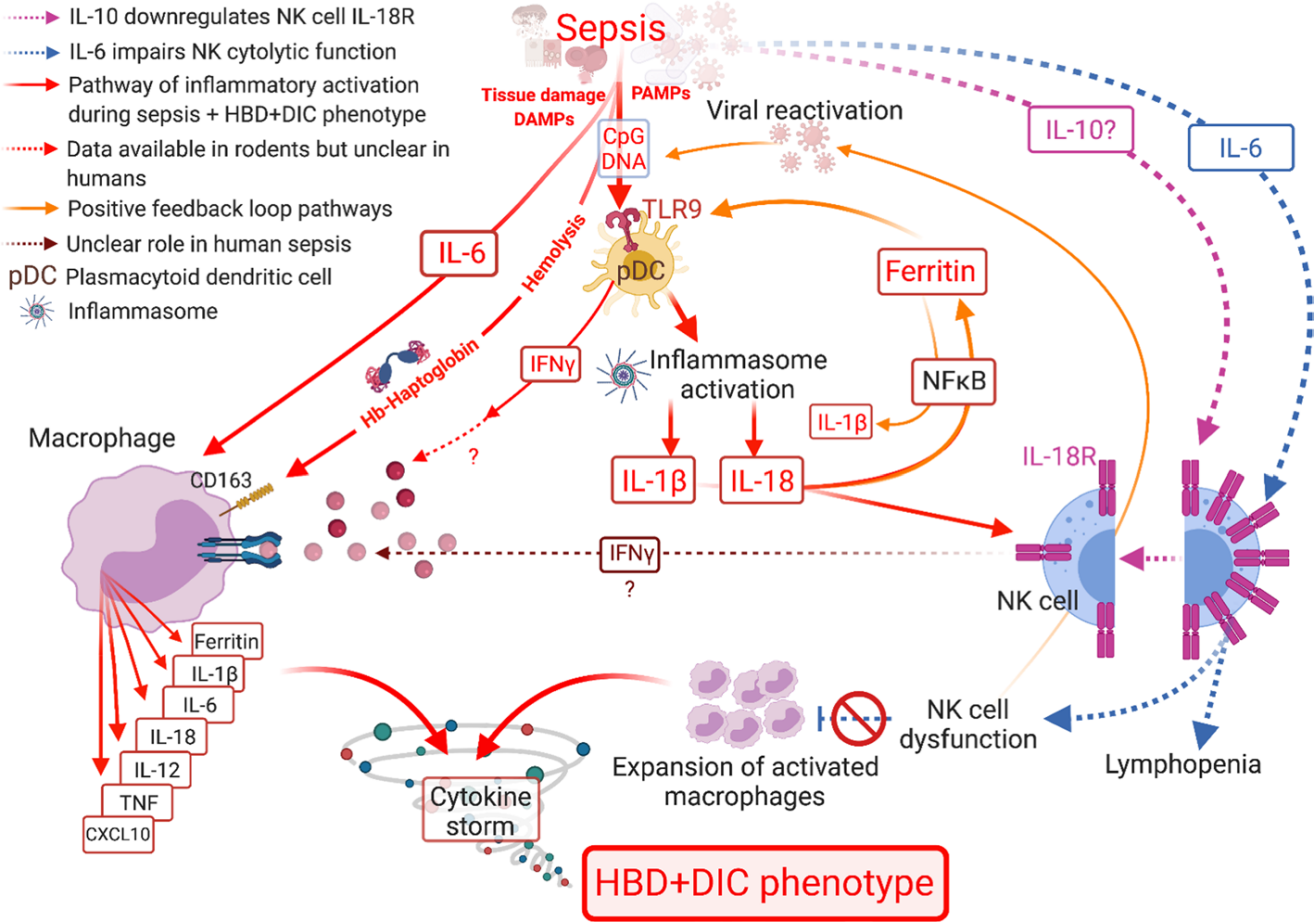
Cell and inflammatory mediator interplay potentially contributes to the development of HBD + DIC in septic patients. Sepsis-induced NK cell deficiency triggers latent viral reactivation, with viral DNA initiating a TLR9-MyD88-mediated signaling cascade [6, 52]. Subsequent inflammasome activation leads to the secretion of IL-18 and IL-1β [6, 56]. In addition to mediating liver injury [59], IL-1β increases transcription and translation of ferritin [60], and production of IL-6 [5]. The release of damage associated molecular patterns (DAMPs), such as mitochondrial DNA or hemoglobin after tissue injury or hemolysis, triggers macrophage activation independent of IFN-γ. Release of free hemoglobin increases hemoglobin-haptoglobin complexes, activating macrophages to produce extracellular ferritin through the CD163 receptor [6, 56]. Ferritin promotes expression of IL-1β and TLR9 [57, 58], resulting in a positive feedback loop with amplification of inflammatory signals [56]. IL-18, in combination with a secondary signal, such as IL-12 or TLR ligands, activates NK cells to produce IFN-γ [53, 64]. We hypothesize, though, that in the context of sepsis with HBD + DIC, NK cells are limited in their responsiveness to IL-18 due to IL-10-mediated downregulation of the IL-18R [55]. As a result, circulating IFN-γ levels are reduced. However, IL-6 enhances signaling through TLRs, increasing the secretion of proinflammatory mediators, including CXCL10 [5]. Persistent NK cell cytolytic dysfunction, stemming from decreased cell number [52] and high levels of IL-6 [54], translates into an impaired ability to induce apoptosis of activated macrophages [6]. In addition, inflammatory mediators produced by macrophages reinforce macrophage (ferritin, IL-6, IL-1β, IL-12, TNF), pDC (ferritin), and lymphocyte (IL-18, IL-12, CXCL10) activation. Thus, the inflammatory cycle continues unabated (cytokine storm), resulting in organ dysfunction and death in the absence of appropriate therapies. The role of pDC- and NK cell-derived IFN-γ in macrophage activation during sepsis remains unclear. In our study, IFN-γ levels were low, although two of its downstream mediators, CXCL10 and IL-18BP, were elevated in patients with sepsis and HBD + DIC

We next investigated the impact of HBD + DIC on biomarker expression in sepsis and whether this biomarker profile was consistent with MAS-like inflammation. Hierarchical clustering of the panel demonstrated marked differences in expression signatures between septic shock patients with and without HBD + DIC (Additional file 1: Fig. S2). Quantitatively, 21 of the 26 biomarkers were significantly different between the two groups (Fig. 2; Additional file 1: Table S3). Relative to sepsis controls, patients with HBD + DIC were characterized by increased expression of ferritin, IL-18, sCD163, sCD25, IL-6, and CXCL10 (Fig. 2; Additional file 1: Table S3), consistent with a biomarker signature of macrophage activation (Additional file 1: Table S1). We also found increased circulating concentrations of IL-18BP and IL-10 in the HBD + DIC cohort (Fig. 2; Additional file 1: Table S3), suggesting the involvement of both pro- and anti-inflammatory soluble factors. Moreover, IL-10 levels were positively correlated with ferritin (*ρ* = 0.22, *p* = 0.047) and IL-18 (*ρ* = 0.33, *p* = 0.002) in the HBD + DIC subset but not in sepsis controls (ferritin: *ρ* = 0.06, *p* = 0.57; IL-18: *ρ* = 0.16, *p* = 0.167). Overall, the biomarker pattern associated with the HBD + DIC phenotype closely resembled that reported in association with other MAS subsets (Additional file 1: Table S1), and the most notable differences in patients with HBD + DIC relative to sepsis controls were among biomarkers that have been associated mechanistically with the hyperinflammatory pathophysiology observed in MAS, including ferritin, IL-18, IL-6, and CXCL10 [5] (Fig. 3).

### *Chronic liver disease does not alter the biomarker signature or the risk of death in HBD* + *DIC*

A limitation of using liver SOFA scores as the HBD criterion for the identification of sepsis patients with HBD + DIC is the inability to distinguish between acute and chronic liver disease. In the matched cohort, chronic liver disease was more prevalent in patients with HBD + DIC than in controls (Table 1; OR = 11.9, 95% CI 4.1–32.4, *p* < 0.001). Therefore, we investigated whether the biomarker signature and risk of death at 90 days were influenced by the presence of chronic liver disease in patients with HBD + DIC. Chronic liver disease did not affect biomarker expression, with the exception of higher ferritin and M-CSF in patients with HBD + DIC in the absence of chronic liver disease (data not shown). In addition, HBD + DIC patients with and without chronic liver disease had similar 90-day mortality (58% vs 55%, *p* = 0.82), and HBD + DIC remained an independent risk factor for death at 90 days after including chronic liver disease as a covariate in the multivariate logistic regression model (*n* = 163) (OR = 2.9, 95% CI = 1.2–7.2, *p* = 0.018). These results were not surprising given the shared histopathologic and inflammatory features between new onset and chronic HBD [44–46], suggesting that, irrespective of cause or eliciting factors, the biomarker signature and mortality related to the HBD + DIC phenotype are driven by this particular pattern of multiple organ dysfunction.

### *The macrophage activation biomarker panel is highly predictive of HBD* + *DIC and mortality*

**Fig. 4:**
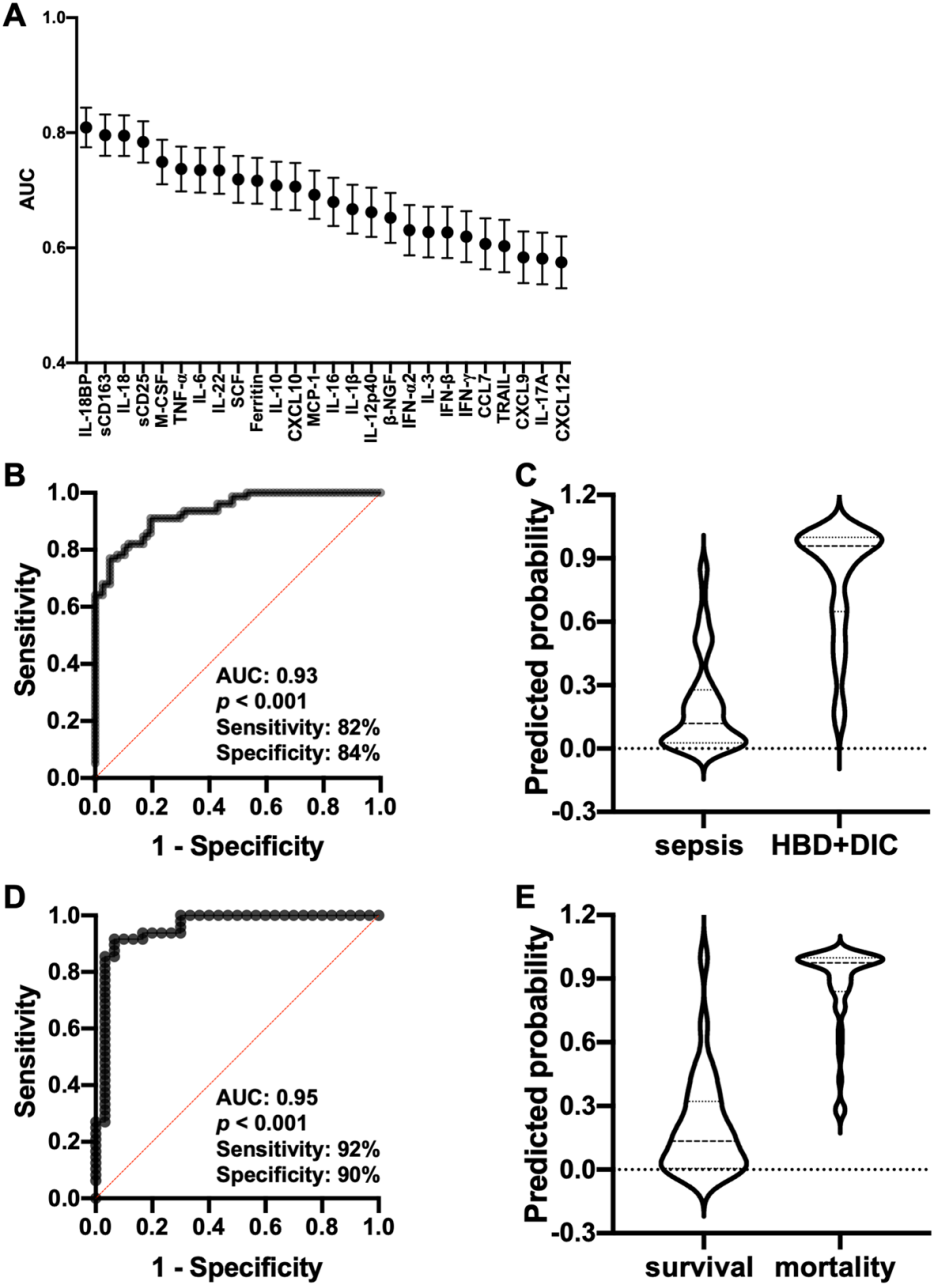
Performance of selected biomarkers to predict the presence of HBD + DIC and 90-day mortality during sepsis. **A** AUC for each of the 26 biomarkers to predict the presence of HBD + DIC in patients with sepsis, organized from highest to lowest. **B** ROC curve representing the model using 26 biomarkers for predicting the HBD + DIC phenotype in patients with sepsis. **C** Violin plots showing the distribution of predicted probabilities for the presence of HBD + DIC in sepsis. The model performed well at classifying both sepsis controls and sepsis with HBD + DIC. The majority of sepsis controls had predicted probabilities of presenting with HBD + DIC less than 0.50 (median: 0.12, IQR: 0.03–0.28). By contrast, the cases had predicted probabilities that were overwhelmingly greater than 0.50 (median: 0.96, IQR: 0.65–0.99). **D** ROC curve representing the model using 26 biomarkers for predicting 90-day mortality among the HBD + DIC subset of patients with sepsis. **E** Violin plots showing the distribution of predicted probabilities for mortality in the cases. The model performed well at distinguishing between survivors (median: 0.13, IQR: 0.01–0.32) and non-survivors (median: 0.97, IQR: 0.84–0.99) among those with HBD + DIC

We examined which of the 26 biomarkers best discriminated HBD + DIC patients in the setting of septic shock. The biomarker with the highest area under the ROC curve (AUC) for predicting the occurrence of HBD + DIC was IL-18BP (AUC = 0.81, 95% CI 0.74–0.88), followed by sCD163 (AUC = 0.80, 95% CI 0.73–0.87), IL-18 (AUC = 0.79, 95% CI 0.73–0.86), and sCD25 (AUC = 0.78, 95% CI 0.71–0.85) (Fig. 4A; Additional file 1: Table S4). A model including these four biomarkers predicted HBD + DIC (AUC = 0.86, 95% CI 0.81–0.92, *p* < 0.001), but the inclusion of all 26 proved highly predictive of HBD + DIC, with an AUC of 0.93 (95% CI 0.90–0.97, *p* < 0.001) and sensitivity and specificity of 82% and 84% (Fig. 4B, C). Notably, this macrophage activation-associated biomarker panel was powerful at discriminating between survivors and non-survivors among the HBD + DIC subset of patients with sepsis (Fig. 4D, E; AUC = 0.95, 95% CI 0.90–1.00, *p* < 0.001, sensitivity = 92%, specificity = 90%).

## Discussion

In accordance with prior reports [13, 16], HBD + DIC was present in approximately 6% of patients with septic shock. The HBD + DIC phenotype was associated with higher mortality and significantly increased expression of key biomarkers implicated in macrophage activation and MAS. Importantly, the macrophage activation-targeted biomarker panel was highly predictive of the HBD + DIC phenotype and mortality.

Chronic liver disease, renal impairment, and cancer were more frequently present among the HBD + DIC sepsis patients, all of which have potential pathophysiologic links to the development of HBD + DIC. First, patients with sepsis and multiple organ failure (MOF) who develop any of three inflammatory pathobiology phenotypes are characterized by an increased proclivity to develop concomitant HBD + DIC and higher mortality. These inflammatory phenotypes include thrombocytopenia associated MOF, defined by new thrombocytopenia (or DIC) and renal dysfunction; immune paralysis associated MOF, defined by immune suppression (i.e., low WBC count); and sequential MOF [14]. Second, MAS commonly arises as a complication of rheumatic conditions, infections, and, importantly, malignancies [5, 47, 48]. Third, numerous features of chronic liver disease predispose to sepsis with HBD + DIC, including a high incidence of bacterial infections (e.g., spontaneous bacterial peritonitis), chronic systemic inflammation [46], and the profound immune dysregulation inherent to chronic liver injury [49, 50]. Fourth, the histopathology of liver tissue from patients with HLH/MAS is consistent with chronic hepatitis [44, 45], suggesting shared features of acute and chronic liver injury that promote organ failure and mortality in the absence of appropriate therapies. Indeed, our data support this pathophysiologic association, as we demonstrated that the HBD + DIC phenotype was more likely to be observed in septic shock patients with chronic liver disease.

Although IFN-γ is a critical driver of the hyperinflammatory state in the spectrum of HLH syndromes [22], it is unclear how IFN-γ contributes to the hyperinflammatory phenotype in sepsis patients with HBD + DIC. HBD + DIC cases exhibited an increase in IFN-γ compared with sepsis controls, but 84% of the HBD + DIC cohort had levels that fell within the normal reference range [51]. The median was also up to 50-fold lower than values reported in association with MAS or sHLH [22, 51]. Despite this, the IFN-γ-stimulated mediators CXCL10 and IL-18BP were increased in patients with HBD + DIC, indicating that IFN-γ signaling may still play a role in the development of this phenotype. Moreover, lower levels of IFN-γ in sepsis-induced HBD + DIC compared to other forms of MAS are not surprising given the effect of sepsis on NK cells. In addition to causing lymphopenia [6, 52], sepsis impairs NK cell cytotoxicity and IFN-γ responses to IL-18 [53, 54]. This mechanism appears to be mediated by IL-10, as serum IL-10 levels negatively correlate with IL-18 receptor (IL-18R) expression on liver NK cells, with neutralization of IL-10 restoring IL-18R expression and IFN-γ responses [55]. In line with these preclinical data, we noted a significant positive correlation between IL-10 and IL-18 in patients with HBD + DIC. Therefore, the counterintuitive pattern of increased IL-18 with only modest elevations in IL-18-induced IFN-γ in the HBD + DIC cohort may be explained by IL-10-mediated downregulation of the IL-18R on NK cells.

Two IFN-γ-independent pathways are also capable of triggering macrophage activation in sepsis. Hemolysis causes an increase in hemoglobin–haptoglobin complexes, which activate macrophages through the CD163 receptor to produce extracellular ferritin [6, 56]. Ferritin increases TLR9 expression and promotes NF-κB-dependent production of IL-1β [57, 58], a proinflammatory cytokine mediating liver injury [59]. Second, sepsis-induced lymphopenia not only dampens host apoptosis of activated macrophages but also contributes to the reactivation of latent viruses [6], initiating a signaling cascade through TLR9 that leads to inflammasome activation and production of IL-18 and IL-1β [6, 56], as well as extracellular ferritin [60], with repeated TLR9 stimulation leading to the clinical appearance of a cytokine storm [19]. Importantly, chronic IL-18 elevation strongly correlates with MAS risk, and excess free IL-18 promotes severe experimental MAS [34]. These events promote a positive feedback loop with amplification of inflammation and liver injury. Thus, we propose that NK cells do not mediate the inflammatory HBD + DIC phenotype in sepsis through IFN-γ but rather that persistent NK cell cytolytic dysfunction, stemming from a quantitative reduction in cell number and high levels of IL-6 [52, 54], translates into an impaired ability to induce apoptosis of activated macrophages [6].

The role of inflammasome activity in macrophage activation indicates HBD + DIC patients may benefit from therapeutic modalities targeting IL-18 and/or IL-1β. Total IL-18 and IL-18BP are typically elevated in sepsis-induced HBD + DIC, albeit to a lesser extent than in MAS [1, 6], but the increase in both suggests IL-18 has bioactive effects. Although increased circulating IL-1β levels in the HBD + DIC cohort appear biologically irrelevant, IL-1β contributes to a feedforward proinflammatory amplification loop even at low concentrations [61]. Furthermore, a causative role for IL-1β has been shown in clinical trials using recombinant IL-1 receptor antagonists to inhibit IL-1 signaling, effectively reducing mortality from 65 to 35% in patients with sepsis and HBD + DIC [13]. Only inhibition of both IL-18 and IL-1β completely protects against mortality in a murine model of lethal endotoxemia [62], and combined blockade of IL-18 and IL-1β has been successfully implemented in a genetic form of MAS resulting from NLRC4 inflammasome hyperactivity [63]. These data suggest that simultaneous neutralization of both IL-1β and IL-18 could have additive value over blocking either cytokine alone in the treatment of HBD + DIC.

## Limitations

Matching of patients with HBD + DIC to controls occurred only based on age and septic shock, potentially increasing the risk of confounding variables. However, our results were robust to sensitivity analyses, suggesting that HBD + DIC is a distinct phenotype in sepsis. The panel of biomarkers tested was incomplete and biased toward those previously associated with MAS/hyperinflammation, and not all analytes had the best dynamic range. Finally, as this was a retrospective analysis, we were unable to test anti-cytokine therapies, but our findings substantiate the trialing of MAS-targeted therapeutics to improve outcomes in septic shock patients with HBD + DIC.

## Conclusion

Concomitant HBD + DIC is a simple clinical strategy to identify septic shock patients who portray biomarker features of macrophage activation and progress with high mortality. This represents a key subgroup of patients to target for future trials testing treatments for macrophage activation and MAS.

## Supplementary Information

**Additional file 1: Figure S1. A.** Relative distribution of sepsis controls and HBD + DIC sepsis cases within each treatment arm of the ProCESS trial. Arm 1 refers to protocol-based early goal directed therapy, Arm 2 to protocol-based standard therapy not requiring placement of a central venous catheter, and Arm 3 to usual care [17]. **B.** Unadjusted in-hospital and 90-day mortality in sepsis controls vs sepsis with HBD + DIC. **C.** Unadjusted 90-day mortality in sepsis controls and sepsis with HBD + DIC, stratified according to treatment arm, with comparisons in mortality drawn between resuscitation strategies within controls and cases. Statistical significance was determined by Fisher’s Exact test. ***p < 0.001, ns = not significant. **Figure S2.** Heatmap demonstrating the hierarchical clustering of 26 macrophage activation-associated biomarkers after forced clustering of columns by group. Biomarkers were measured in samples collected within 30 min of admission to the emergency department (time 0 h).

## Data Availability

The data sets used and/or analyzed during the current study are available from the corresponding author on reasonable request.

## Abbreviations

APACHE: Acute Physiology and Chronic Health Evaluation
AUC: Area under the ROC Curve
DAMP: Damage Associated Molecular Pattern
DIC: Disseminated Intravascular Coagulation
HBD: Hepatobiliary Dysfunction
IL: Interleukin
IL-18R: IL-18 Receptor
INR: International Normalized Ratio
IQR: Interquartile Range
IU: International Units
MAS: Macrophage Activation Syndrome
MOF: Multiple Organ Failure
ROC Curve: Receiver Operating Characteristic Curve
sHLH: Secondary Hemophagocytic Lymphohistiocytosis
SOFA: Sequential Organ Failure Assessment
WBC: White Blood Cell
