# Supplementary material for: Sepsis with liver dysfunction and coagulopathy predicts an inflammatory pattern of macrophage activation
Source: Intensive Care Med Exp. 2022 Feb 21;10:6. doi: 10.1186/s40635-022-00433-y (PMC8861227; doi:10.1186/s40635-022-00433-y)
Supplement: Supplementary file 1 — Additional file 1: Figure S1. A. Relative distribution of sepsis controls and HBD + DIC sepsis cases within each treatment arm of the ProCESS trial. Arm 1 refers to protocol-based early goal directed therapy, Arm 2 to protocol-based standard therapy not requiring placement of a central venous catheter, and Arm 3 to usual care [17]. B. Unadjusted in-hospital and 90-day mortality in sepsis controls vs sepsis with HBD + DIC. C. Unadjusted 90-day mortality in sepsis controls and sepsis with HBD + DIC, stratified according to treatment arm, with comparisons in mortality drawn between resuscitation strategies within controls and cases. Statistical significance was determined by Fisher’s Exact test. ***p < 0.001, ns = not significant. Figure S2. Heatmap demonstrating the hierarchical clustering of 26 macrophage activation-associated biomarkers after forced clustering of columns by group. Biomarkers were measured in samples collected within 30 min of admission to the emergency department (time 0 h). [file 40635_2022_433_MOESM1_ESM.docx]

**Supplementary Table of Contents**

[Tables 2](file:///C:\Users\0016236\AppData\Roaming\Jobs\40635\433\author\Supplemental%20Material.docx#_Toc92895012)

[Table S1. Biomarkers with a role in macrophage activation 2](file:///C:\Users\0016236\AppData\Roaming\Jobs\40635\433\author\Supplemental%20Material.docx#_Toc92895013)

[Table S2. Summary of missing data 4](file:///C:\Users\0016236\AppData\Roaming\Jobs\40635\433\author\Supplemental%20Material.docx#_Toc92895014)

[Table S3. Biomarker characteristics of sepsis controls and HBD+DIC 5](file:///C:\Users\0016236\AppData\Roaming\Jobs\40635\433\author\Supplemental%20Material.docx#_Toc92895015)

[Table S4. AUC for each biomarker for predicting HBD+DIC 6](file:///C:\Users\0016236\AppData\Roaming\Jobs\40635\433\author\Supplemental%20Material.docx#_Toc92895016)

[Figures 7](file:///C:\Users\0016236\AppData\Roaming\Jobs\40635\433\author\Supplemental%20Material.docx#_Toc92895017)

[Figure S1. Distribution and mortality of sepsis cases and controls by treatment arm 7](file:///C:\Users\0016236\AppData\Roaming\Jobs\40635\433\author\Supplemental%20Material.docx#_Toc92895018)

[Figure S2. Hierarchical clustering heatmap of 26 macrophage activation-associated biomarkers 8](file:///C:\Users\0016236\AppData\Roaming\Jobs\40635\433\author\Supplemental%20Material.docx#_Toc92895019)

[Appendix 9](file:///C:\Users\0016236\AppData\Roaming\Jobs\40635\433\author\Supplemental%20Material.docx#_Toc92895020)

[The ProCESS Investigators 9](file:///C:\Users\0016236\AppData\Roaming\Jobs\40635\433\author\Supplemental%20Material.docx#_Toc92895021)

# **Tables**

## **Table S1.** **Biomarkers with a role in macrophage activation**

| **Biomarker** | **References** | **Study Setting** | | **Finding** |
| --- | --- | --- | --- | --- |
| IL-1β | Shakoory 2016 | Human | | IL-1 receptor antagonist is associated with reduced mortality risk in sepsis patients with features of MAS |
| IL-6 | Billiau 2005 | Human | | In situ expression of IL-6 and TNF-α by hemophagocytosing macrophages on liver tissues of patients with MAS |
| IL-10 | Behrens 2011 | Murine | | Blockade of IL-10 signaling leads to fulminant MAS |
| IL-17A | Jovanovic 1998 | Primary human MDMs | | Sustains inflammatory responses by stimulating secretion of IL-1β, IL-6, and TNF-α |
| IFN-γ | Bracaglia 2017 | Human | | Circulating levels significantly higher in active sJIA with MAS than active sJIA without MAS |
| CXCL10 | Bracaglia 2017 | Human | | Circulating levels significantly higher in active sJIA with MAS and active HLH |
| MCP-1 | Tamura 2008 | Human | | Elevated MCP-1 levels precede increases in serum ferritin in active HLH |
| TNF-α | Billiau 2005 | Human | | In situ expression of IL-6 and TNF-α by hemophagocytosing macrophages on liver tissues of patients with MAS |
| sCD25 | Bleesing 2007 | Human | | Increased circulating levels in MAS associated with sJIA |
| IFN-α2 | Menten 1999 | Human PBMCs | | Promotes production of CCL7 protein in human mononuclear cells in a dose-dependent manner |
| CXCL12 | Sanchez-Martin 2011 | Primary human monocytes | | Monocyte CXCL12 secretion leads to differentiation to CD163^+^ macrophages in an autocrine/paracrine manner |
| CCL7 | Xuan 2015 | Primary human MDMs | | Induces chemotaxis of both M1 and M2 macrophages |
| β-NGF | Samah 2008 | Primary human MDMs | | Increases CXCR4 expression on macrophages and chemotactic responses to suboptimal levels of CXCL12 |
| TRAIL | Cartland 2019 | Human | | Inverse correlation between plasma TRAIL and IL-18, with reduced TRAIL expression potentially promoting macrophage accumulation and inflammation |
| SCF | Heyworth 1992 | Murine bone marrow | | Combination of SCF with M-CSF promotes  the development of macrophages |
| IL-16 | Mathy 2000 | Human PBMCs | | Stimulates production of IL-1β, IL-6, and TNF-α by CD14^+^CD4^+^ monocytes and maturing macrophages |
| IL-3 | Weber 2015 | Murine | | Indirectly mediates induction of  IL-1β, IL-6, and TNF-α in sepsis |
| IL-12p40 | Osugi 1997;  Canna 2014 | Human | | Elevated plasma concentrations in pediatric HLH;  component of NLRC4-MAS signature |
|  |  |  | |  |
| **Table S1 (continued)** | | |  |  |
| **Biomarker** | **References** | **Study Setting** | | **Finding** |
| M-CSF | Akashi 1994;  Canna 2014 | Human | | Extremely high serum concentrations in  adult patients with active HLH;  component of NLRC4-MAS signature |
| IL-22 | Weber 2007 | Murine | | Blockade of IL-22 attenuates expression of IL-10, IL-6, and TNF-α and reduces sepsis-associated bacterial load and renal failure |
| sCD163 | Bleesing 2007 | Human | | Increased circulating levels in MAS associated with sJIA |
| IFN-β | Menten 1999 | Human PBMCs | | Promotes production of CCL7 protein in human mononuclear cells in a dose-dependent manner |
| IL-18 | Mazodier 2005;  Weiss 2018;  Canna 2014 | Human | | Severe imbalance of IL-18 in MAS; IL-18 distinguishes and pathogenically promotes MAS; component of NLRC4-MAS signature |
| IL-18BP | Mazodier 2005  Weiss 2018 | Human | | Moderate elevation of IL-18BP, resulting in excess biologically active free IL-18 |
| CXCL9 | Bracaglia, 2017 | Human | | Circulating levels significantly higher in  active sJIA with MAS and active HLH |
| Ferritin | Ravelli 2005  Ravelli 2016  Fardet 2014 | Human | | Extreme hyperferritinemia is a defining feature of MAS |
| HLH, hemophagocytic lymphohistiocytosis; MAS, macrophage activation syndrome; MDMs, monocyte-derived macrophages; PBMCs, peripheral blood mononuclear cells; sJIA, systemic juvenile idiopathic arthritis. | | | | |

## **Table S2. Summary of missing data**

| **Clinical Characteristics** | | | **Biomarkers** | | |
| --- | --- | --- | --- | --- | --- |
| **% Missing** | **Sepsis Controls** | **HBD+DIC** | **% Missing** | **Sepsis Controls** | **HBD+DIC** |
| Age | 0 | 0 | IL-1β | 2.4 | 1.2 |
| Gender | 0 | 0 | IL-6 | 2.4 | 1.2 |
| Race | 0 | 0 | IL-10 | 2.4 | 1.2 |
| Ethnicity | 0 | 0 | IL-17A | 2.4 | 1.2 |
| WBC | 0 | 0 | IFN-γ | 2.4 | 1.2 |
| Bilirubin | 8.5 | 0 | CXCL10 | 2.4 | 1.2 |
| Platelets | 0 | 0 | MCP-1 | 2.4 | 1.2 |
| Creatinine | 1.2 | 0 | TNF-α | 2.4 | 1.2 |
| INR | 18.3 | 0 | sCD25 | 2.4 | 1.2 |
| All SOFA Scores | 0 | 0 | IFN-α2 | 2.4 | 1.2 |
| APACHE III | 0 | 0 | CXCL12 | 2.4 | 1.2 |
| Mechanical Ventilation | 0 | 0 | CCL7 | 2.4 | 1.2 |
| Charlson Index | 0 | 0 | β-NGF | 2.4 | 1.2 |
| Hypertension | 0 | 0 | TRAIL | 2.4 | 1.2 |
| Myocardial Infarction | 0 | 0 | SCF | 2.4 | 1.2 |
| Cong Heart Failure | 0 | 0 | IL-16 | 2.4 | 1.2 |
| Chronic Resp Dz | 0 | 0 | IL-3 | 2.4 | 1.2 |
| Cerebral Vasc Dz | 0 | 0 | IL-12p40 | 2.4 | 1.2 |
| Peripheral Vasc Dz | 0 | 0 | M-CSF | 2.4 | 1.2 |
| Diabetes Mellitus | 0 | 0 | IL-22 | 6.1 | 3.7 |
| Chronic Liver Dz | 0 | 0 | sCD163 | 6.1 | 3.7 |
| Renal Impairment | 0 | 0 | IFN-β | 6.1 | 3.7 |
| AIDS | 0 | 0 | IL-18 | 2.4 | 1.2 |
| Cancer | 0 | 0 | IL-18BP | 2.4 | 1.2 |
| In-hospital Mortality | 0 | 0 | CXCL9 | 2.4 | 1.2 |
| 90-day Mortality | 0 | 0 | Ferritin | 2.4 | 2.4 |
| APACHE, acute physiology and chronic health evaluation; Cong, congestive; Dz, disease; HBD+DIC, sepsis with hepatobiliary dysfunction and disseminated intravascular coagulation; INR, international normalized ratio; Resp, respiratory; SOFA, sequential organ failure assessment; Vasc, vascular; WBC, white blood cells. | | | | | |

## **Table S3.** **Biomarker characteristics of sepsis controls and HBD+DIC**

|  | **Sepsis Controls** | | **HBD+DIC** | | ***p* value*** |
| --- | --- | --- | --- | --- | --- |
| **Biomarker** | **Median (*n*)** | **IQR** | **Median (*n*)** | **IQR** |  |
| IL-1β (pg/ml) | 3.19 (*80*) | 2.76–3.88 | 3.88 (*81*) | 3.19–7.19 | 0.003 |
| IL-6 (pg/ml) | 22.2 (*80*) | 13.9–107 | 250 (*81*) | 30.2–2319 | 0.003 |
| IL-10 (pg/ml) | 44.3 (*80*) | 38.8–58.9 | 84.4 (*81*) | 46.5–182 | 0.003 |
| IL-17A (pg/ml) | 43.5 (*80*) | 41.3–48.6 | 46.1 (*81*) | 43.3–53.2 | 0.189 |
| IFN-γ (pg/ml) | 13.3 (*80*) | 12.4–14.2 | 14.2 (*81*) | 12.6–16.1 | 0.049 |
| CXCL10 (ng/ml) | 0.98 (*80*) | 0.41–3.31 | 3.44 (*81*) | 1.24–29.5 | 0.003 |
| MCP-1 (pg/ml) | 95.3 (*80*) | 60.5–210 | 252 (*81*) | 103–2394 | 0.003 |
| TNF-α (pg/ml) | 28.2 (*80*) | 19.7–40.7 | 62.6 (*81*) | 31.1–180 | 0.003 |
| sCD25 (pg/ml) | 396 (*80*) | 336–564 | 687 (*81*) | 445–1339 | 0.003 |
| IFN-α2 (pg/ml) | 121 (*80*) | 115–131 | 129 (*81*) | 118–142 | 0.035 |
| CXCL12 (pg/ml) | 277 (*80*) | 235–327 | 288 (*81*) | 246–337 | 0.189 |
| CCL7 (pg/ml) | 197 (*80*) | 180–218 | 205 (*81*) | 192–232 | 0.087 |
| β-NGF (pg/ml) | 45.0 (*80*) | 39.6–51.7 | 50.1 (*81*) | 44.0–57.1 | 0.007 |
| TRAIL (pg/ml) | 39.0 (*80*) | 33.9–51.8 | 35.3 (*81*) | 31.4–42.0 | 0.090 |
| SCF (pg/ml) | 123 (*80*) | 105–168 | 171 (*81*) | 139–230 | 0.003 |
| IL-16 (pg/ml) | 1056 (*80*) | 851–1477 | 1411 (*81*) | 1047–1725 | 0.003 |
| IL-3 (pg/ml) | 741 (*80*) | 693–789 | 787 (*81*) | 709–846 | 0.039 |
| IL-12p40 (pg/ml) | 769 (*80*) | 699–936 | 872 (*81*) | 776–1164 | 0.003 |
| M-CSF (pg/ml) | 32.5 (*80*) | 26.8–42.5 | 46.8 (*81*) | 35.9–85.1 | 0.003 |
| IL-22 (pg/ml) | 49.1 (*77*) | 44.1–58.2 | 59.3 (*79*) | 53.1–83.5 | 0.003 |
| sCD163 (ng/ml) | 196 (*77*) | 134–291 | 514 (*79*) | 280–828 | 0.003 |
| IFN-β (pg/ml) | 39.1 (*77*) | 37.2–42.7 | 42.0 (*79*) | 39.1–44.7 | 0.041 |
| IL-18 (pg/ml) | 333 (*80*) | 251–425 | 656 (*81*) | 424–1402 | 0.003 |
| IL-18BP (ng/ml) | 5.28 (*80*) | 3.13–10.7 | 18.6 (*81*) | 10.6–31.3 | 0.003 |
| CXCL9 (ng/ml) | 3.15 (*80*) | 1.90–7.02 | 4.63 (*81*) | 2.13–13.8 | 0.189 |
| Ferritin (ng/ml) | 215 (*80*) | 64.6–455 | 482 (*80*) | 240–1516 | 0.003 |
| HBD+DIC, sepsis with hepatobiliary dysfunction and disseminated intravascular coagulation; IQR, interquartile range.  The number of patients included in each biomarker analysis is indicated next to the median (of *n* = 82 cases and controls).  *adjusted *p* value in comparing sepsis controls with HBD+DIC | | | | | |

## **Table S4. AUC for each biomarker for predicting HBD+DIC**

| **Biomarker** | **AUC** | **Std Error** | **95% CI** | ***p* value** |
| --- | --- | --- | --- | --- |
| IL-18BP | 0.809 | 0.035 | 0.742–0.877 | <0.001 |
| sCD163 | 0.796 | 0.036 | 0.726–0.866 | <0.001 |
| IL-18 | 0.794 | 0.035 | 0.726–0.864 | <0.001 |
| sCD25 | 0.784 | 0.036 | 0.713–0.854 | <0.001 |
| M-CSF | 0.749 | 0.039 | 0.673–0.825 | <0.001 |
| TNF-α | 0.737 | 0.039 | 0.660–0.814 | <0.001 |
| IL-6 | 0.735 | 0.039 | 0.659–0.811 | <0.001 |
| IL-22 | 0.734 | 0.040 | 0.655–0.813 | <0.001 |
| SCF | 0.719 | 0.041 | 0.639–0.799 | <0.001 |
| Ferritin | 0.717 | 0.040 | 0.638–0.795 | <0.001 |
| IL-10 | 0.708 | 0.041 | 0.627–0.789 | <0.001 |
| CXCL10 | 0.706 | 0.041 | 0.626–0.787 | <0.001 |
| MCP-1 | 0.692 | 0.042 | 0.610–0.774 | <0.001 |
| IL-16 | 0.680 | 0.042 | 0.597–0.762 | <0.001 |
| IL-1β | 0.667 | 0.043 | 0.584–0.750 | 0.001 |
| IL-12p40 | 0.662 | 0.043 | 0.578–0.746 | 0.001 |
| β-NGF | 0.652 | 0.043 | 0.567–0.737 | 0.001 |
| IFN-α2 | 0.631 | 0.044 | 0.545–0.716 | 0.004 |
| IL-3 | 0.627 | 0.044 | 0.542–0.713 | 0.005 |
| IFN-β | 0.627 | 0.045 | 0.539–0.714 | 0.006 |
| IFN-γ | 0.620 | 0.044 | 0.533–0.706 | 0.009 |
| CCL7 | 0.607 | 0.044 | 0.520–0.694 | 0.019 |
| TRAIL | 0.603 | 0.045 | 0.514–0.692 | 0.024 |
| CXCL9 | 0.584 | 0.045 | 0.495–0.672 | 0.067 |
| IL-17A | 0.582 | 0.045 | 0.494–0.670 | 0.074 |
| CXCL12 | 0.575 | 0.045 | 0.487–0.663 | 0.101 |
| AUC, area under the ROC curve; CI, confidence interval; HBD+DIC, sepsis with hepatobiliary dysfunction and disseminated intravascular coagulation; std error,  standard error. | | | | |

# **Figures**

## **Figure S1. Distribution and mortality of sepsis cases and controls by treatment arm**


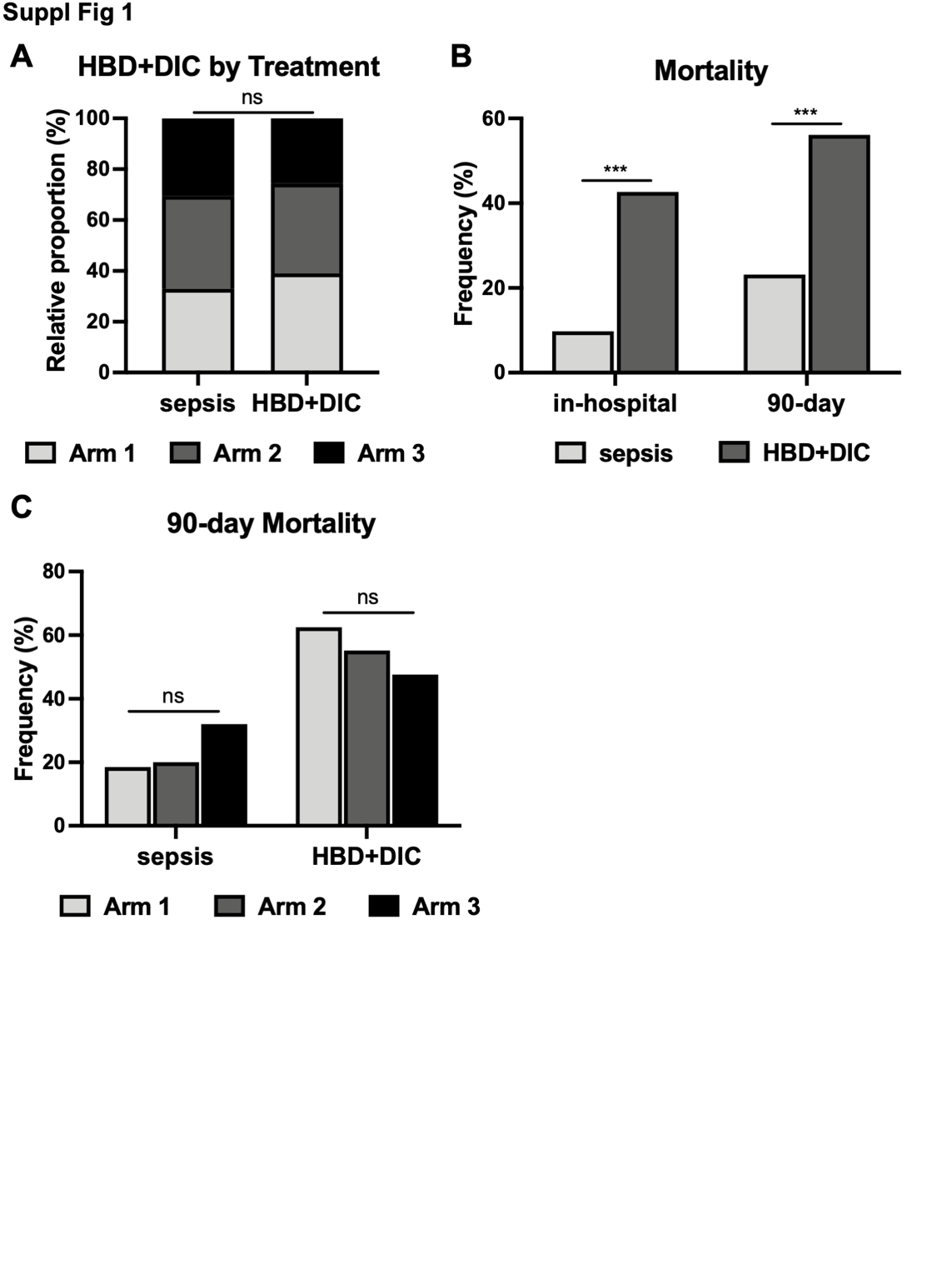


## **Figure S2. Hierarchical clustering heatmap of 26 macrophage activation-associated biomarkers**

**
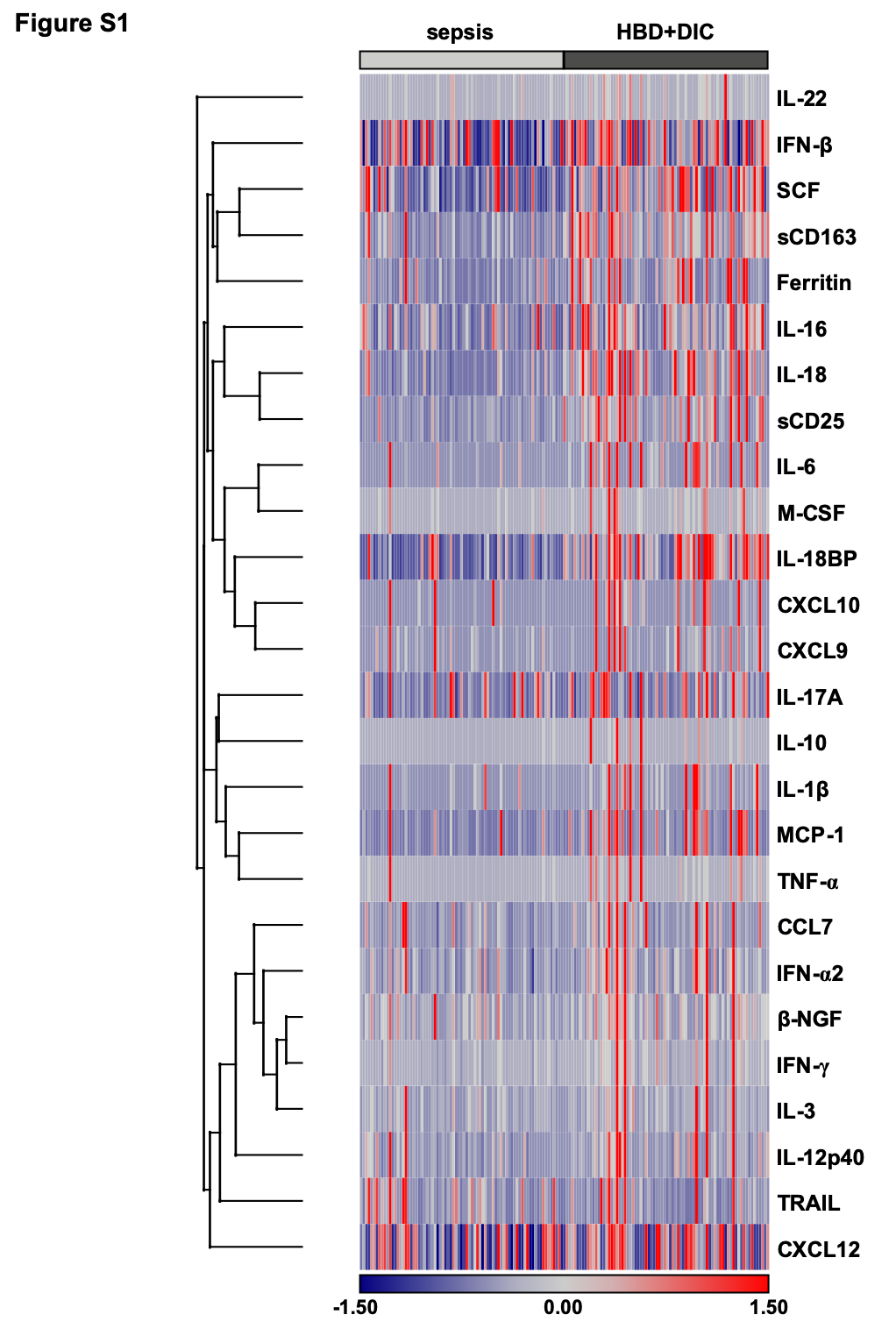
**

# **Appendix**

## **The ProCESS Investigators**

The members of the ProCESS Trial are as follows: Coordinating Center: Derek C. Angus, Amber E. Barnato, Tammy L. Eaton, Elizabeth Gimbel, David T. Huang, Christopher Keener, John A. Kellum, Kyle Landis, Francis Pike, Diana K. Stapleton, Lisa A. Weissfeld, Michael Willochell, Kourtney A. Wofford, Donald M. Yealy. Recruiting Centers: (Site Principal Investigators are listed in Italics) - Advocate Christ Medical Center, Oak Lawn, IL - Erik Kulstad, Hannah Watts. Allegheny General Hospital, Pittsburgh, PA - Arvind Venkat. Brigham and Women’s Hospital, Boston, MA - Peter C. Hou, Anthony Massaro, Siddharth Parmar. Duke University Medical Center, Durham, NC - Alexander T. Limkakeng, Jr. East Carolina University, Greenville, NC - Kori Brewer, Theodore R. Delbridge, Allison Mainhart. George Washington University Medical Center, Washington, DC - Lakhmir S. Chawla. Hennepin County Medical Center, Minneapolis, MN - James R. Miner. Intermountain Medical Center, Murray, UT - Todd L. Allen, Colin K. Grissom, Los Angeles County + USC Medical Center, Los Angeles, CA - Stuart Swadron. Louisiana State University Health Sciences Center, Shreveport, LA - Steven A. Conrad. Maricopa Medical Center, Phoenix, AZ - Richard Carlson, Frank LoVecchio. Massachusetts General Hospital, Boston, MA - Ednan K. Bajwa, Michael R. Filbin. Blair A. Parry. Methodist Research Institute, Indianapolis, IN - Timothy J. Ellender. North Shore University Hospital, Manhasset, NY - Andrew E. Sama. Norwalk Hospital, Norwalk, CT - Jonathan Fine. Penn State Hershey College of Medicine, Hershey, PA - Soheil Nafeei, Thomas Terndrup, Margaret Wojnar. Stanford University School of Medicine, Stanford, CA - Ronald G. Pearl. Summa Health System, Akron, OH - Scott T. Wilber. SUNY Downstate Medical Center, Brooklyn, NY - Richard Sinert. Tampa General Hospital, Tampa, FL - David J. Orban, Jason W. Wilson. Temple University Hospital, Philadelphia, PA - Jacob W. Ufberg. UC Davis Medical Center, Sacramento, CA - Timothy Albertson, Edward A. Panacek. University Medical Center Brackenridge, Austin, TX - Sohan Parekh. UPMC Presbyterian/Shadyside, Pittsburgh, PA - Scott R. Gunn, Jon S. Rittenberger, Richard J. Wadas. University of Alabama at Birmingham, Birmingham, AL - Andrew R. Edwards, Matthew Kelly, Henry E. Wang, University of Arkansas for Medical Sciences, Little Rock, AR - Talmage M. Holmes. University of Maryland at Baltimore, Baltimore, MD - Michael T. McCurdy. University of Minnesota Medical Center, Fairview, MN - Craig Weinert. University of Utah Health Sciences Center, Salt Lake City, UT - Estelle S. Harris. Vanderbilt University Medical Center, Nashville, TN - Wesley H. Self, Diane Dubinski. Washington Hospital Center, Washington, DC - Carolyn A. Phillips, Ronald M. Migues.
